# Relative Contribution of Framework and CDR Regions in Antibody Variable Domains to Multimerisation of Fv- and scFv-Containing Bispecific Antibodies

**DOI:** 10.3390/antib7030035

**Published:** 2018-08-31

**Authors:** Pallavi Bhatta, David P. Humphreys

**Affiliations:** Protein Sciences Group, UCB Pharma, Slough, Berkshire SL1 3WE, UK; David.Humphreys@ucb.com

**Keywords:** bispecific antibody, disulphide stabilised Fv, disulphide stabilised single chain Fv, monomer, thermal stability

## Abstract

Bispecific antibodies represent an emerging class of antibody drugs that are commonly generated by fusion of Fv or scFv antigen binding domains to IgG or Fab scaffolds. Fv- or scFv-mediated multimerisation of bispecific antibodies via promiscuous vH-vL pairing can result in sub-optimal monomer levels during expression, and hence, undesirable therapeutic protein yields. We investigate the contribution of disulphide stabilised Fv and scFv to Fab-Fv and Fab-scFv multimerisation. We show that monomer levels of isolated Fv/scFv cannot always be used to predict monomer levels of Fab-linked Fv/scFv, and that Fab-scFv monomer levels are greater than the equivalent Fab-Fv. Through grafting bispecifics with framework/CDR-‘swapped’ Fv and scFv, we show that monomer levels of disulphide stabilised Fab-Fv and Fab-scFv can be improved by Fv framework ‘swapping’. The Fab-Fv and Fab-scFv can be considered representative of the significant number of bispecific antibody formats containing appended Fv/scFv, as we also used Fv framework ‘swapping’ to increase the monomer level of an IgG-scFv bispecific antibody. This research may, therefore, be useful for maximising the monomeric yield of numerous pharmaceutically-relevant bispecific formats in pre-clinical development.

## 1. Introduction

**Figure 1:**
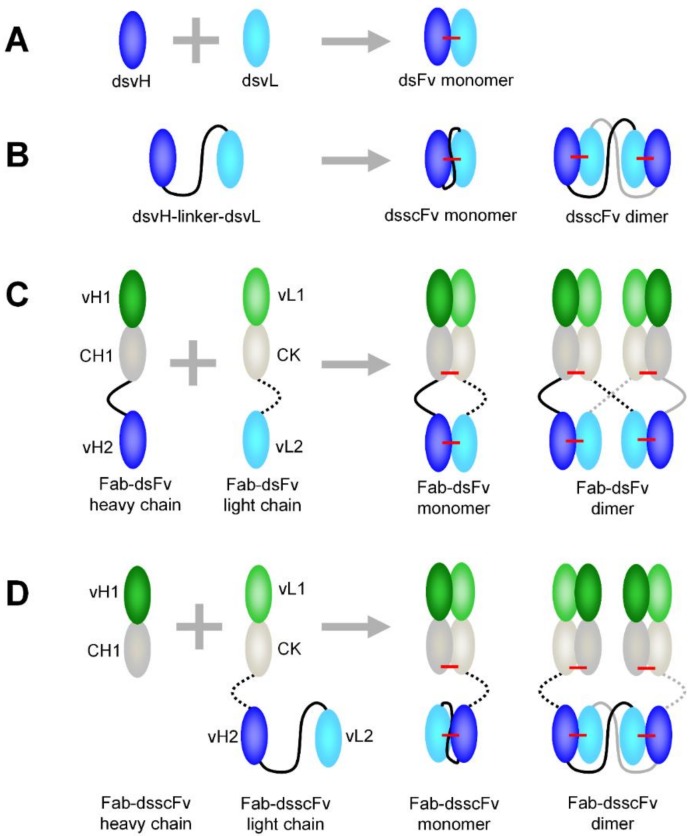
Potential dimerisation mechanisms of isolated and Fab-linked dsFv and dsscFv during cellular expression. (**A**) dsFv; (**B**) dsscFv; (**C**) Fab-dsFv; (**D**) Fab-dsscFv. Disulphide bonds are shown in red. During cellular expression, dsvH and dsvL can pair correctly to form disulphide stabilised monomers. ‘Mispairing’ of dsvH and dsvL can result in unwanted disulphide stabilised dimers (and higher order multimers). Monomers and multimers do not interchange, as the disulphide bond within the dsFv/dsscFv fixes the monomer:multimer ratio, allowing purification of the desired monomeric species. Although Fab appended formats are shown, similar vH-vL ‘mispairing’ can occur in IgG formats appended with dsFv/dsscFv.

Through simultaneous binding to two antigens, bispecific antibodies can invoke synergistic or novel biology and may offer enhanced clinical efficacy via improved drug targeting. Fv and scFv are discrete antigen binding domains that have been fused to Fab or IgG scaffolds to confer multispecificity in a wide variety of formats (reviewed in [1]). Conversion of Fv to scFv through introduction of a polypeptide linker between the vH and vL was originally carried out to stabilise the relatively weak vH-vL interface, but dynamic domain exchange (‘breathing’) between proximal scFv monomers can result in variable levels of monomer, dimer and higher-order multimers [2]. The introduction of a disulphide (ds) bond in isolated scFv between the vH and vL prevents variable domain ‘breathing’ and thus fixes monomer:multimer ratios [2]. The result of disulphide stabilised vH and vL ‘mispairing’ between proximal scFv monomers (in isolated scFv or scFv-containing bispecific antibodies), is a spectrum of dimer, trimer and higher order species. Formation of a vH-vL disulphide bond irreversibly locks all multimeric forms during expression/purification, and therefore enables the separation of desirable (monomeric) from undesirable (multimeric) species by purification. In isolated disulphide stabilised scFv (dsscFv), the presence of a linker enables stable dimer/multimer formation (Figure 1B). Conversely, isolated disulphide stabilised Fv (dsFv) might be expected to form 100% monomer, as there is no linker available to connect monomers together (Figure 1A). Fab or IgG fusion proteins appended with a dsFv may however have additional complexities. Figure 1C,D illustrates potential dimerisation mechanisms of formats appended with dsFv or dsscFv, demonstrating how inappropriate multimerisation can occur during cellular expression. 

The term ‘mispairing’ in the context of this paper is defined as that resulting from the intermolecular pairing of a dsvH in one intended dsFv/dsscFv on a carrier molecule, with a dsvL appended to a different carrier molecule of the same type. Antigen binding is retained in the resulting multimers owing to the fact that the vH-vL pairs are all ‘appropriate’. However, multimers may have unwanted biological properties (e.g., receptor crosslinking, avidity effects) as well as concentration/storage issues, and must therefore be removed during subsequent bioprocessing steps. High levels of multimer formation during protein expression will thus ultimately result in low overall therapeutic protein yields. 

We compare Fab-dsFv [3,4] and Fab-dsscFv as exemplar Fv- and scFv-containing bispecific antibodies to investigate the influence of dsFv versus dsscFv on bispecific antibody monomer level, owing to the molecular simplicity compared to IgG appended formats. The Fab-dsFv can be considered to be a model for bispecific formats where vH and vL are appended off neighbouring C-terminii such as IgG(H)-Fv [5,6], whilst the Fab-dsscFv may be considered to be representative of formats containing appended scFv such as IgG(H)-scFv and IgG(L)-scFv [7,8,9,10,11,12]. We further investigate the relative influence on multimerisation of dsFv and dsscFv framework (FW) and CDR residues through grafting a range of Fv FW/CDR ‘swapped’ Fab-dsFv and Fab-dsscFv. We also analyse the effect of the framework and CDR residues on Fv thermal stability to assess whether there is a relationship between thermal stability and monomer level. Lastly, we analyse the effect of Fv framework ‘swapping’ in an IgG-dsscFv bispecific antibody. 

## 2. Materials and Methods

### 2.1. Reagents

All materials, reagents and human embryonic kidney (HEK) 293F cell lines were sourced from Life Technologies (Paisley, UK) unless otherwise stated. 

### 2.2. Plasmid Construction

The Fab-dsFv light chain is constructed as signal peptide-vL1-cK-Ser(Gly_4_Ser)_3_-dsvL2, where vL1 is the humanised variable light domain 1, cK is the human kappa light chain constant domain and vL2 is the humanised variable light domain 2. The Fab-dsFv heavy chain is constructed as signal peptide-vH1-CH1-Ser(Gly_4_Ser/Thr)_3_-dsvH2, where vH1 is the humanised variable heavy domain 1, CH1 is the human gamma-1 heavy chain CH1 constant domain and vH2 is the humanised variable heavy domain 2. The Fab-dsscFv light chain is constructed as signal peptide-vL1-cK-Ser(Gly_4_Ser)_2_-dsvH2-(Gly_4_Ser)_4_-dsvL2. The Fab-dsscFv heavy chain is constructed as signal peptide-vH1-CH1. Isolated dsscFv were expressed in HL orientation and constructed as signal peptide-dsvH-(Gly_4_Ser)_4_-dsvL-10xHis, where 10xHis is a C-terminal epitope tag. Isolated dsFv were expressed as signal peptide-dsvH and signal peptide-dsvL-10xHis from separate plasmids. Linker sequences are listed here for clarification: Fab-dsFv light chain linker (SGGGGSGGGGSGGGGS), Fab-dsFv heavy chain linker (SGGGGSGGGGTGGGGS), Fab-dsscFv light chain linker (SGGGGSGGGGS), scFv linkers connecting vH2 and vL2 (GGGGSGGGGSGGGGSGGGGS). All isolated and Fab-linked Fv and scFv were disulphide stabilised, containing cysteines at vH44-vL100 (Kabat numbering). Proteins were expressed from one of two closely related CMV-containing UCB-modified mammalian expression plasmids; pNAFL was used for cloning and expression of light chain constructs while pNAFH was used for cloning and expression of heavy chain constructs and scFv. The two plasmids possess largely identical sequences and have been observed to have very similar functionalities. 

### 2.3. Antibody Expression

Expression plasmids were co-transfected (1:1 ratio of heavy:light chain) into 293F cells using 293fectin™ transfection reagent according to the manufacturer’s instructions. The cells were cultured in FreeStyle™ media with shaking at 37 °C. Cell culture supernatants were harvested 10 days post-transfection by centrifugation at 2000 rpm for 10 min. Supernatants were clarified by passage through a 0.22 μm filter. 

### 2.4. Octet Quantification Assay

dsFv and dsscFv protein concentration in the cell culture supernatant was determined using an Octet (ForteBio) and software version 4.0.7. Penta-His biosensors were prepared according to the manufacturer’s instructions and supernatants analysed using the following parameters: assay time 120 s, shake speed 200 rpm, dip and read time 120 s. A standard curve was generated using a purified His-tagged dsscFv (UCB) at a concentration of 1–100 µg/mL using a dose-response (4PL) equation. All standards and samples were read in duplicate. Octet limit of detection = 5 µg/mL.

### 2.5. Immobilised Metal Affinity Chromatography (IMAC)

Batch Ni^2+^-NTA purification was carried out as described previously [2]. Briefly, Ni^2+^-NTA Superflow resin (Qiagen, Manchester, UK) was mixed gently with pH and salt-adjusted cell culture supernatant at 4 °C overnight. The resin was washed three times with 50 mM phosphate, 300 mM NaCl, 40 mM imidazole, pH8.0, followed by elution with 50 mM phosphate, 300 mM NaCl, 250 mM imidazole, pH8.0. The eluates were filtered through a 1 μm syringe filter and then concentrated and buffer exchanged into phosphate-buffered saline (PBS), pH7.4, using an Amicon Ultra-4, 3 kDa molecular weight cut-off membrane centrifuge tube. 

### 2.6. Protein G and Protein A HPLC Purification

Fab-dsFv, Fab-dsscFv and IgG(H)-dsscFv protein concentration in the cell culture supernatant was determined by a protein G HPLC assay by comparison of the A_280_ signal to a Fab standard. The 293F supernatants were concentrated ~25 fold using an Amicon Ultra-15, 10 kDa (Fab-dsFv, Fab-dsscFv) or 30 kDa (IgG(H)-dsscFv) molecular weight cut-off membrane centrifugation concentrator. Fab-dsFv and Fab-dsscFv concentrated supernatants were applied to a 1 mL HiTrap Protein-G column (GE Healthcare, Little Chalfont, UK) equilibrated in 20 mM phosphate, 40 mM NaCl, pH7.4. The column was washed with 20 mM phosphate, 40 mM NaCl, pH7.4, and the bound material was eluted with 0.1 M glycine/HCl, pH2.7. IgG(H)-dsscFv concentrated supernatants were applied to a 1 mL HiTrap MabSelect Protein-A column (GE Healthcare, Little Chalfont, UK) equilibrated in 20 mM phosphate, 40 mM NaCl, pH7.4. The column was washed with 20 mM phosphate, 40 mM NaCl, pH7.4, and the bound material was eluted with 0.1 M sodium citrate, pH3.4. Protein G and Protein A eluates were collected and pH-adjusted to ~pH 7.0 with 2 M Tris/HCl, pH8.5. The pH-adjusted eluate was concentrated and buffer exchanged into PBS, pH7.4, using a 10 kDa (Fab-dsFv, Fab-dsscFv) or 30 kDa (IgG(H)-dsscFv) molecular weight cut-off centrifugation concentrator. 

### 2.7. SDS-PAGE

Purified protein samples (5 μg) containing 1× NuPAGE^®^ LDS sample buffer and either 1× NuPAGE^®^ sample reducing agent (reduced samples) or 10 mM N-Ethylmaleimide (NEM, ThermoFisher Scientific, Loughborough, UK) (non-reduced samples) were incubated at 70 °C for 10 min. The samples were run on a 4–20% Tris-glycine gel in Tris-Glycine SDS buffer after which protein bands were detected using Instant Blue gel stain (Expedeon, Cambridge, UK). SeeBlue^TM^ Plus2 pre-stained protein standard (ThermoFisher Scientific, Loughborough, UK) was used as a marker. 

### 2.8. ELISA

Flat-bottomed 96 well Nunc MaxiSorp plates were coated with 2 µg/mL antigen in PBS overnight at 4 °C. Plates were blocked with 1% (*w/v*) Polyethylene glycol (PEG, MW 20 kDa, BDH) for 1 h at room temperature, then washed 3× with PBS + 0.1% Tween-20 (PBST). Following incubation with tripling dilutions of purified protein (starting at 1 µg/mL) in 0.1% (*w/v*) PEG, PBST for 1 h at room temperature, plates were washed 3× with PBST and then incubated with HRP-conjugated anti-human cK antibody (SB81a, Southern Biotech, Birmingham, AL, USA) for 1 h at room temperature. Following 3× PBST washes, binding was detected by incubation with 3,3′,5,5′-tetramethylbenzidine (TMB) substrate (Merck, Nottingham, UK). Absorbance was read at 630 nm on a Biotek PowerWave HT Microplate Spectrophotometer (BioTek, Swindon, UK). 

### 2.9. Size Exclusion Chromatography (SEC)

20 µg purified protein sample (100 μL of 0.2 mg/mL stock diluted in PBS, pH7.4) was injected onto either a Superdex 200 10/300 GL Tricorn column (GE Healthcare, Little Chalfont, UK) or a TSK Gel G3000SWXL, 7.8 × 300 mm, column (Tosoh Bioscience, Reading, UK) 3 days post-purification and developed respectively with an isocratic gradient of PBS, pH 7.4 at 1 mL/min or 200 mM phosphate, pH 7.0 at 1 mL/min. Signal detection was by absorbance at 280 nm. Gel filtration protein standards (BioRad, Watford, UK) were loaded for molecular weight estimation (chromatograms are shown in Supplementary Figure S1). Since all purified fusion proteins contain disulphide stabilised Fv/scFv, the observed monomer level is independent of protein concentration and unaffected by on-column dilution effects. 

### 2.10. Differential Scanning Fluorimetry

Thermal stability analysis was carried out as described previously [2,13]. Briefly, samples contained 3× SYPRO^®^Orange dye and 0.1 mg/mL purified protein in PBS, pH 7.4. The mixture was dispensed in quadruplicate into a 384 PCR optical well plate, which was run on a 7900 HT fast real-time PCR System (Agilent Technologies, Stockport, UK). The PCR system heating device was set at 20 °C to 99 °C with a ramp rate of 1.1 °C/min; a charge coupled device monitored fluorescence changes in the wells. Intensity increase was plotted, and the inflection point of the slope(s) was used to generate the thermal stability transition midpoint (Tm). A higher Tm denotes a more stable protein domain. Thermograms are shown in Supplementary Figure S2.

## 3. Results

### 3.1. Comparison of dsFv and dsscFv

A range of human vK1 vH3 variable region pairs with vH44:vL100 disulphide stabilisation was expressed transiently as C-terminally His-tagged dsFv or dsscFv in 293F cells. We used the vK1 vH3 sub-group, which is routinely used for humanisation and is thus considered to be a model v-region framework pairing. The scFv used in this study are in the HL orientation, whereby the C-terminus of the vH is connected to the N-terminal of the vL via a flexible peptide linker. We expressed the scFv in the HL orientation as we have previously observed higher monomer levels for these variable domain sequences in the HL orientation compared to the LH orientation. We used a 20 amino acid 4×G_4_S linker to connect the vH and vL as these long linkers have been shown to be effective at minimising non-covalent multimerisation [9,14]. This is an important consideration in this study since we wanted to witness the importance of the variable domain sequences in the multimerisation process rather than those driven by any linker constraints. We used the vH44:vL100 disulphide bond position as this has previously been shown to be a preferred disulphide bond location that is well tolerated amongst different scFv and Fv [2,9,15].

**Figure 2:**
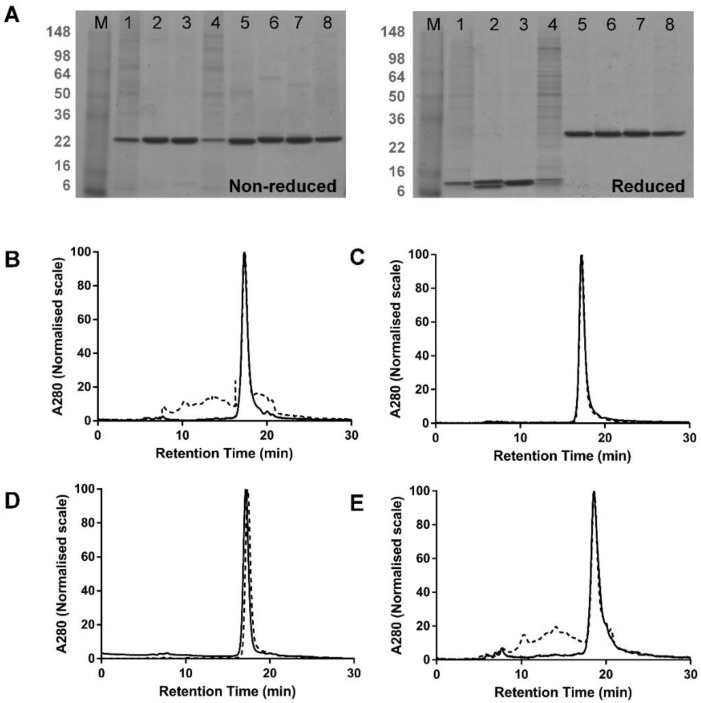
Multimerisation of dsFv vs. dsscFv. (**A**) Non-reducing and reducing SDS-PAGE analysis of dsFv and dsscFv proteins IMAC purified from 293F cells—representative data from one of three independent experiments are shown. M—molecular weight marker; lane 1—dsFv#1; lane 2—dsFv#2; lane 3—dsFv#3; lane 4—dsFv#4; lane 5—dsscFv#1; lane 6—dsscFv#2; lane 7—dsscFv#3; lane 8—dsscFv#4. (**B**–**E**) S200 SEC profiles of purified dsFv and dsscFv proteins. (**B**) dsFv/dsscFv#1; (**C**) dsFv/dsscFv#2; (**D**) dsFv/dsscFv#3; (**E**) dsFv/dsscFv#4. Dashed lines represent dsFv; continuous lines denote dsscFv. Peak heights are normalised to 100%. Gel filtration protein standards (BioRad) were loaded for molecular weight estimation.

Four dsFv and four dsscFv proteins were expressed transiently in 293F cells. The variable domain primary sequences in dsFv#1, dsFv#2, dsFv#3 and dsFv#4 are the same as those used respectively in dsscFv#1, dsscFv#2, dsscFv#3 and dsscFv#4. Fv#1-4 bind to three distinct target antigens: Fv#2 is specific for a serum protein, whilst the target antigens for Fv#1, Fv#3 and Fv#4 are all soluble cytokines. Fv#3 and Fv#4 bind to the same target antigen, but possess unique sequences and were discovered independently. Following expression, the cell supernatants were analysed by Octet using Penta-His tips. It has been suggested that expression of dsFv without a linker is unattainable or extremely inefficient in mammalian cells and can only be achieved in bacteria through periplasmic expression or refolding of cytoplasmic inclusion bodies [5]. This is thought to be due to the weak association between vL and vH [16] resulting in inherently poor hetero-pairing. We successfully expressed four different dsFv in 293F cells, as judged by SDS-PAGE post-purification (Figure 2A), although the expression levels of dsFv#1 and dsFv#4 in the cell supernatant were below the limit of detection by Octet measurement. The expression levels of the dsFv were comparatively lower than the corresponding dsscFv, with dsFv#2 and dsFv#3 expression levels (29.4 μg/mL and 37.6 μg/mL) being respectively ~50% and 80% that of the equivalent dsscFv (Table 1). To put these yields in context, Fab and IgG expressed in 293F cells using the same plasmids and signal peptide, gave expression levels of 32.6 μg/mL and 39.8 μg/mL, respectively. 

The dsFv and dsscFv proteins were purified from the cell culture supernatant by a batch Ni^2+^-NTA method and the purified proteins were analysed by non-reducing and reducing SDS-PAGE (Figure 2A) and SEC (Figure 2B–E). 

SDS-PAGE analysis revealed prominent high molecular weight species in dsFv#1 and dsFv#4 samples (Figure 2A, lanes 1 and 4 respectively), which were also the two poorest expressing dsFv. These bands remained under reducing conditions and may represent host cell proteins that have co-purified with these dsFv. These bands are also visible in other samples, albeit to a much lower extent and were not detected by anti-His immune-blotting (data not shown). SEC analysis showed that dsFv#2 and dsFv#3 were 99–100% monomeric, whereas dsFv#1 and dsFv#4 appeared to display lower monomer levels of 40–44% (Table 1). Again, in the absence of a linker, it is improbable that this low monomer level represents multimeric dsFv and more likely reflects contamination by host cell proteins. Thus, dsFv#1 and dsFv#4 are also likely to be 100% monomeric. All dsscFv were highly monomeric, with monomer levels of 97.0%, 100.0%, 99.5% and 93.4% respectively for dsscFv#1, dsscFv#2, dsscFv#3 and dsscFv#4. 

The thermal stabilities (Tm’s) of the purified dsFv and dsscFv proteins, determined by differential scanning fluorimetry, are displayed in Table 1. The Tm’s of the dsscFv were towards the upper end of published scFv stabilities and were broadly comparable with their dsFv counterparts. The presence of the scFv linker appears to have marginally destabilised scFv#3, as the Tm of dsscFv#3 (61.2 °C) was several degrees lower than dsFv#3 (66.8 °C). Perhaps this represents the kind of steric constraint which could be alleviated with even longer linkers [11] or expression in the LH orientation. Similarly, the Tm of scFv#1 (57.8 °C) was slightly lower than dsFv#1 (59.5 °C). Conversely, the scFv linker appears to have stabilised dsFv#2 as the Tm of scFv#2 (76.9 °C) was slightly higher than dsFv#2 (75.2 °C). All dsFv and dsscFv displayed a single Tm, except dsFv#1 and dsFv#4, which displayed an additional minor unfolding transition at ~52 °C, which likely represents host cell protein contaminants. 

### 3.2. Comparison of Fab-dsFv and Fab-dsscFv

The same dsFv or dsscFv (minus His-tag) was then fused to the C-terminal end of HER2 (4D5) Fab, which has been well characterised in the literature and has been shown to be 100% monomeric [17]. We made Fab(LC)-dsscFv proteins, as opposed to Fab(HC)-dsscFv, because the Fab(LC)-dsscFv constructs were readily available. Following transient expression in 293F cells, the concentration of Fab-dsFv and Fab-dsscFv proteins in the culture supernatant was determined by a Protein G HPLC assay. The expression level of the Fab-dsFv ranged from 15.1–28.1 μg/mL, whereas the expression level of the Fab-dsscFv in the cell supernatant was somewhat lower at 15.8–21.8 μg/mL (Table 2), although there was one exception where the expression level of Fab-dsscFv#4 (21.8 μg/mL) was higher than Fab-dsFv#4 (15.1 μg/mL). 

**Figure 3:**
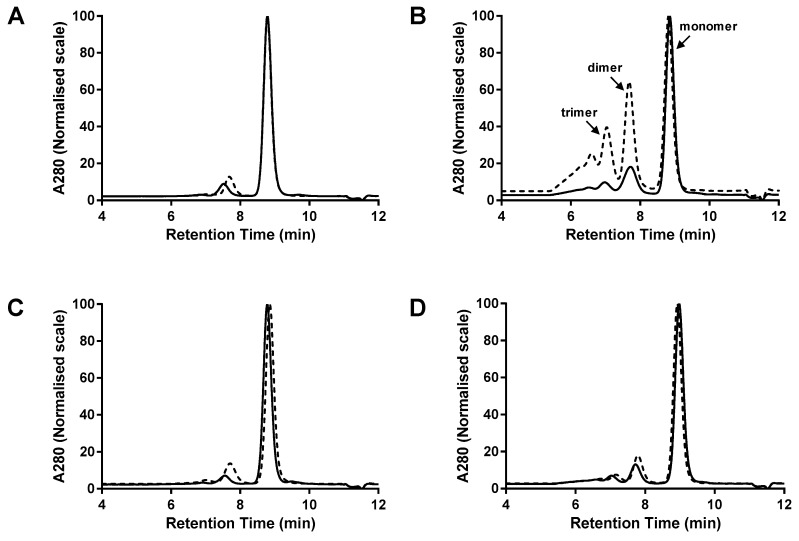
Multimerisation of Fab-dsFv vs. Fab-dsscFv. G3000 SEC analysis of proteins purified by Protein G HPLC from 293F cells – representative data from one of three independent experiments are shown. (**A**) Fab-dsFv/dsscFv#1; (**B**) Fab-dsFv/dsscFv#2; (**C**) Fab-dsFv/dsscFv#3; (**D**) Fab-dsFv/dsscFv#4. Dashed lines represent Fab-dsFv; continuous lines denote Fab-dsscFv. Peak heights are normalised to 100%. Gel filtration protein standards (BioRad) were loaded for molecular weight estimation.

Proteins were purified from the cell culture supernatant by Protein G HPLC and the purified proteins were analysed by SEC (Figure 3). Since Protein G binds to the CH1 domain, any light chain dimers that may have formed during expression will be absent from the purified material. Differential monomer levels were observed for Fab-dsFv and Fab-dsscFv proteins comprising equivalent sequences in the Fv position, with monomer levels of the Fab-dsscFv being higher in all four examples than the equivalent Fab-dsFv. 

In the three examples where the Fab-dsFv were inherently highly monomeric (Fab-dsFv#1: 87.5% monomer, Fab-dsFv#3: 83.7% monomer, Fab-dsFv#4: 76.0% monomer), conversion of dsFv to dsscFv resulted in only a minor increase in monomer level (Fab-dsscFv#1: 91.2% monomer, Fab-dsscFv#3: 91.8% monomer, Fab-dsscFv#4: 79.9% monomer) (Table 2, Figure 3A,C,D). However, in the single case where the Fab-dsFv had a very low monomer level (Fab-dsFv#2), the monomer level was almost doubled from 37.8% to 70.9% by conversion of the Fab-dsFv to a Fab-dsscFv (Table 2, Figure 3B). Isolated dsFv#2/dsscFv#2 appeared to be 100% monomeric, so the inherent tendency of Fv#2 to multimerise only becomes apparent when the dsFv or dsscFv is fused to a Fab. Thus, there appears to be no correlation of monomer level in free dsFv/dsscFv formats to that of the related Fab-dsFv/Fab-dsscFv formats, indicating that ‘free intermolecular association’ properties are not the same as ‘tethered intermolecular association’ properties. 

Thermal stabilities of the Fab-dsFv and Fab-dsscFv proteins were determined by differential scanning fluorimetry (Table 2). The isolated HER2 Fab has a Tm of 80.7 °C, and this was reduced by ~2 °C by fusion of dsFv/dsscFv. There was close correlation between the Fv Tm from dsFv/dsscFv and Fab-dsFv/Fab-dsscFv. For example, the dsFv Tm’s of dsFv#2 and Fab-dsFv#2 respectively were 75.2 °C and 72.4 °C and the dsscFv Tm’s of dsscFv#2 and Fab-dsscFv#2 respectively were 76.9 °C and 73.1 °C. The Fab appended dsFv/dsscFv generally displayed a Tm that was typically a few degrees lower than the isolated dsFv/dsscFv, except in the case of dsscFv#1, where the dsscFv Tm in Fab-dsscFv#1 (59.0 °C) was slightly higher than the isolated dsscFv#1 (57.8 °C), and in dsscFv#3, where the isolated and Fab appended dsscFv had equal Tm’s (61.2 °C and 61.4 °C respectively). Thus, attachment to a Fab domain via a linker does not substantially affect thermal stability of dsFv/dsscFv. 

### 3.3. Analysis of Fab-dsFv and Fab-dsscFv with Framework/CDR-‘Swapped’ Fv

A range of Fab-dsFv and Fab-dsscFv with framework/CDR (FW/CDR) ‘swapped’ dsFv/dsscFv was analysed to investigate which parts of the variable region drive the differential monomer levels, in essence to determine whether the CDRs or acceptor framework dominate these interactions. Swaps were made whereby all six CDRs from the Fv of a ‘high monomer’ Fab-dsFv/dsscFv were grafted onto a ‘low monomer’ Fv framework and *vice versa*, and tested in the context of a Fab-dsFv and Fab-dsscFv as outlined in Table 3. For example, Fab-dsFv(FW#1/CDR#2) consists of Fv#2 CDRs grafted onto the Fv#1 acceptor framework, whereas Fab-dsFv(FW#2/CDR#1) contains Fv#1 CDRs grafted onto the Fv#2 acceptor framework. Framework and CDR regions were defined according to ABM [18]. Fv#1 and Fv#2 were chosen, owing to their ability to drive superior (Fv#1: 87.5–91.2%) and inferior (Fv#2: 37.8–70.9%) Fab-dsFv/dsscFv monomer levels. The Tm’s of dsFv/dsscFv#1 and dsFv/dsscFv#2 also differ significantly (Fv#1: Tm ~ 59 °C versus Fv#2: Tm ~ 73 °C) so the correlation of the dsFv/dsscFv Tm on Fab-dsFv/dsscFv monomer level could also be assessed. Fv#1 and Fv#2 acceptor frameworks comprise different human germline sequences within the vK1 vH3 subgroup and several parental donor residues. The heavy chain sequences of FW#1 and FW#2 differ by 14 amino acids, whilst the light chain frameworks differ by 5 amino acids. CDRH3 was of similar length in both antibodies, and displayed 13% identity. Following transient expression in 293F cells, proteins were purified from the cell culture supernatant by Protein G HPLC. The monomer level and thermal stability of the purified proteins was analysed respectively by SEC and differential scanning fluorimetry. HER2 Fab was purified and analysed alongside as a control. The expression level of HER2 Fab was 32.6 μg/mL. The expression levels of Fab-dsscFv (6.7–28.5 μg/mL) with wild type and ‘swapped’ Fv were somewhat lower than the corresponding Fab-dsFv (10.6-34.0 μg/mL) (Table 3). Grafting CDR#2 onto FW#1 resulted in increased expression (Fab-dsFv: 28.1 μg/mL to 34.0 μg/mL, Fab-dsscFv: 21.7 μg/mL to 28.5 μg/mL) whereas grafting CDR#1 onto FW#2 resulted in decreased expression (Fab-dsFv: 23.4 μg/mL to 10.6 μg/mL, Fab-dsscFv: 20.0 μg/mL to 6.7 μg/mL). 

**Figure 4:**
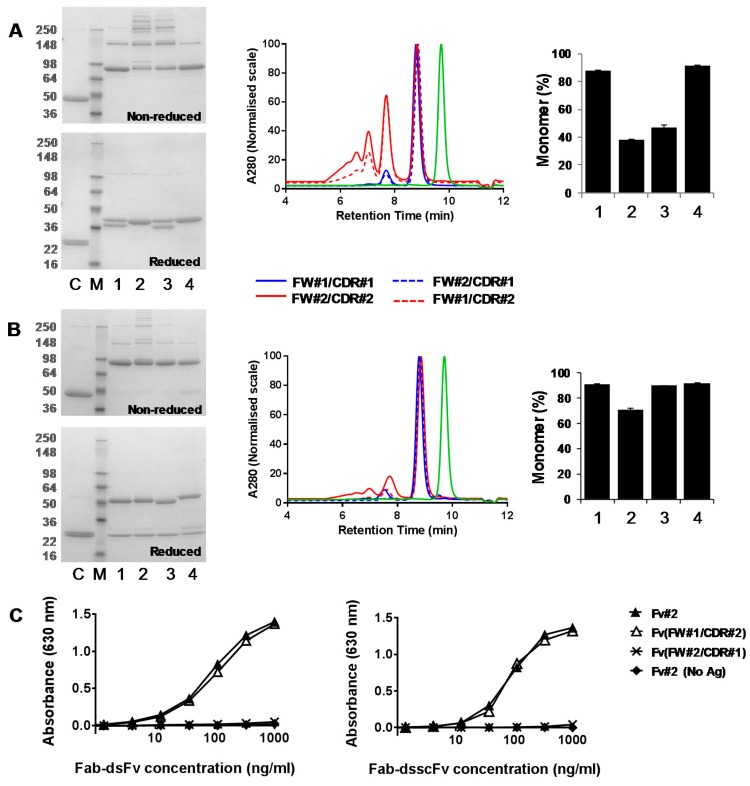
Comparison of Fab-dsFv and Fab-dsscFv with wildtype and FW/CDR-‘swapped’ Fv. SDS-PAGE and G3000 SEC analysis of proteins purified by Protein G HPLC from 293F cells—representative data from one of three independent experiments are shown. (**A**) LEFT—non-reducing and reducing SDS-PAGE gel of Fab-dsFv proteins. M—molecular weight marker; C—HER2 Fab control; lane 1—Fab-dsFv#1; lane 2—Fab-dsFv#2; lane 3—Fab-dsFv(FW#1/CDR#2); lane 4—Fab-dsFv(FW#2/CDR#1). MIDDLE—Fab-dsFv SEC profiles. Blue continuous line—Fab-dsFv#1; red continuous line—Fab-dsFv#2; red dashed line—Fab-dsFv(FW#1/CDR#2); blue dashed line—Fab-dsFv(FW#2/CDR#1); green line—Fab. Gel filtration protein standards (BioRad) were loaded for molecular weight estimation. RIGHT—Bar graph illustrating Fab-dsFv monomer levels. 1—Fab-dsFv#1; 2—Fab-dsFv#2; 3—Fab-dsFv(FW#1/CDR#2); 4—Fab-dsFv(FW#2/CDR#1). Error bars denote mean ± standard deviation (*n* = 3); (**B**) LEFT—non-reducing and reducing SDS-PAGE gel of Fab-dsscFv proteins. M—molecular weight marker; C—HER2 Fab control; lane 1—Fab-dsscFv#1; lane 2—Fab-dsscFv#2; lane 3—Fab-dsscFv(FW#1/CDR#2); lane 4—Fab-dsscFv(FW#2/CDR#1). MIDDLE—Fab-dsscFv SEC profiles. Blue continuous line—Fab-dsscFv#1; red continuous line—Fab-dsscFv#2; red dashed line—Fab-dsscFv(FW#1/CDR#2); blue dashed line—Fab-dsscFv(FW#2/CDR#1); green line—Fab. RIGHT—Bar graph illustrating Fab-dsscFv monomer levels. 1—Fab-dsscFv#1; 2—Fab-dsscFv#2; 3—Fab-dsscFv(FW#1/CDR#2); 4—Fab-dsscFv(FW#2/CDR#1). Error bars denote mean ± standard deviation (*n* = 3); (**C**) Antigen#2 binding ELISA. Filled triangles—Fab-dsFv/dsscFv#2; open triangles—Fab-dsFv/dsscFv(FW#1/CDR#2); crosses—Fab-dsFv/dsscFv(FW#2/CDR#1); filled diamonds—Fab-dsFv/dsscFv#2 (no antigen control).

The reducing SDS-PAGE gels (Figure 4A,B) showed banding patterns which indicated that the constructs were being expressed correctly with bands at ~50 kDa and ~25 kDa (Fab-dsscFv) or a doublet at ~37 kDa (Fab-dsFv). Fab-dsFv with dsFv#2 or dsFv(FW#2/CDR#1) in the Fv position seemingly ran as a single band as the heavy and light chains are almost identical in size (Figure 4A, reduced gel, lanes 2 and 4). There was a small proportion of unreduced protein in all four Fab-dsFv samples under reducing conditions (Figure 4A, reduced gel). There was a small proportion of ‘free’ Fab in all Fab-dsscFv samples, which was most prominent in Fab-dsscFv(FW#2/CDR#1) (Figure 4B, reduced and non-reduced gel, lane 4). 

Percentage monomer data for wild type and ‘swapped’ Fab-dsFv proteins is illustrated in the bar graphs (Figure 4A) and in Table 3. HER2 Fab displayed a high monomer level (98.6%). Fab-dsFv monomer levels appeared to be largely influenced by the Fv CDRs, with those containing dsFv#1 CDRs (‘high % monomer’) in the Fv position always displaying high monomer levels (Fab-dsFv#1: 87.5% monomer, Fab-dsFv(FW#2/CDR#1): 91.6% monomer). In contrast, those containing dsFv#2 (‘low % monomer’) CDRs always displayed low monomer levels regardless of the Fv FW used. An increase in Fab-dsFv monomer level was seen when dsFv#2 CDRs were grafted onto FW#1 (Fab-dsFv(FW#1/CDR#2): 47.1% monomer, compared to Fab-dsFv#2: 37.8% monomer), however this monomer level is still considered to be low in our experience. 

Fv framework ‘swapping’ influenced monomer levels more in the Fab-dsscFv format compared to Fab-dsFv. Percentage monomer data for wild type and ‘swapped’ Fab-dsscFv proteins is illustrated in the bar graphs (Figure 4B) and in Table 3. Grafting dsscFv#2 CDRs onto FW#1 resulted in a high monomer level (Fab-dsscFv(FW#1/CDR#2): 90.1% monomer, compared to Fab-dsscFv#2: 70.9% monomer). The monomer levels of Fab-dsscFv can thus be improved through the grafting of dsscFv CDRs onto a different framework, providing that the antigen affinity is retained within the FW-‘swapped’ dsscFv. In this particular case, essential antigen binding was retained when Fv#2 CDRs were grafted onto FW#1, as shown by antigen-based ELISA (Figure 4C). We have also shown analogous improvements in monomer level in a high density Expi293F cell line. Fab-dsscFv with wild type and framework ‘swapped’ dsscFv showed almost identical monomer levels to those seen in 293F cells, despite significantly higher (4–7 fold) expression levels in Expi293F cells (data not shown). This is an important observation as it shows that monomer level is unaffected by transient expression level, therefore a high-density cell line can be used to maximise expression with no negative influence on monomer level. Fab-dsscFv#1 and Fab-dsscFv(FW#2/CDR#1) showed similarly high monomer levels, although the lower expression and presence of more ‘free’ Fab observed in Fab-dsscFv(FW#2/CDR#1) suggests some instability upon CDR#1 grafting onto FW#2.

To analyse the influence of dsFv framework and CDR sequences on Fab-dsFv thermal stability, the thermograms of wild type and ‘swapped’ Fab-dsFv were compared. Whilst the Tm profiles of Fab-dsFv with a common dsFv framework were quite different, the Tms of Fab-dsFv with common dsFv CDRs were very similar (Table 3, Supplementary Figure S2). This suggests that dsFv thermal stability is driven mainly by CDR residues and not framework residues. Whereas differences were seen between Fab-dsFv and Fab-dsscFv in terms of the contribution of Fv framework and CDR residues to monomer level, the contribution of the Fv CDRs to Tm was the same regardless of whether the Fv was a dsFv or dsscFv. The Fv Tm in ‘swapped’ Fab-dsscFv resembled those of the wild type Fv containing the same CDRs e.g., the Fv Tm in Fab-dsscFv(FW#1/CDR#2) (72.4 °C) was far more similar to that in Fab-dsscFv#2 (73.1 °C) than in Fab-dsscFv#1 (59.0 °C). 

### 3.4. Analysis of IgG(H)-dsscFv with Framework/CDR-‘Swapped’ Fv

**Figure 5:**
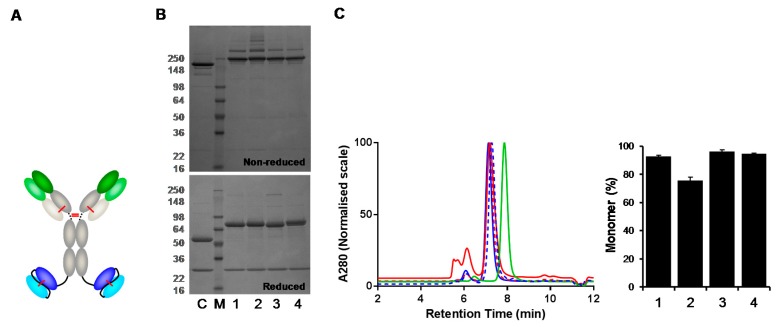
Comparison of IgG(H)-dsscFv with wildtype and FW/CDR-‘swapped’ Fv. SDS-PAGE and G3000 SEC analysis of proteins purified by Protein A HPLC from 293F cells – representative data from one of three independent experiments are shown. (**A**) Structure of IgG(H)-dsscFv. (**B**) Non-reducing and reducing SDS-PAGE gel of IgG(H)-dsscFv proteins. M—molecular weight marker; C—HER2 IgG control; lane 1—IgG(H)-dsscFv#1; lane 2—IgG(H)-dsscFv#2; lane 3—IgG(H)-dsscFv(FW#1/CDR#2); lane 4—IgG(H)-dsscFv(FW#2/CDR#1). (**C**) IgG(H)-dsscFv SEC profiles. Blue continuous line—IgG(H)-dsscFv#1; red continuous line—IgG(H)-dsscFv#2; red dashed line—IgG(H)-dsscFv(FW#1/CDR#2); blue dashed line—IgG(H)-dsscFv(FW#2/CDR#1); green line—IgG. RIGHT—Bar graph illustrating IgG(H)-dsscFv monomer levels. Error bars denote mean ± standard deviation (*n* = 3).

As dsscFv(FW#1/CDR#2) ‘swaps’ were beneficial in Fab-based bispecific formats, we assessed whether CDR#2 grafting onto FW#1 could improve monomer levels of IgG-like bispecific formats. IgG(H)-dsscFv bispecific antibodies containing HER2 IgG with wild type and FW/CDR ‘swapped’ dsscFv were analysed (Figure 5A). Following transient expression in 293F cells, IgG(H)-dsscFv#1, IgG(H)-dsscFv#2, IgG(H)-dsscFv(FW#1/CDR#2) and IgG(H)-dsscFv(FW#2/CDR#1) proteins in the culture supernatant were quantified by Protein G HPLC, and purified from the cell culture supernatant by Protein A HPLC. The monomer level of the purified proteins was analysed by SEC. In addition, HER2 IgG was purified and analysed alongside as a control. The expression level of HER2 IgG was 39.8 μg/mL, whereas IgG(H)-dsscFv expression ranged from 3.7–39.1 μg/mL (Table 4). Improved expression was again seen when dsscFv#2 CDRs were grafted onto FW#1 (IgG(H)-dsscFv(FW#1/CDR#2): 39.1 μg/mL versus IgG(H)-dsscFv#2: 24.9 μg/mL). In accordance with Fab-dsscFv data, decreased expression was seen when dsscFv#1 CDRs were grafted onto FW#2 (IgG(H)-dsscFv(FW#2/CDR#1): 3.7 μg/mL versus IgG(H)-dsscFv#1: 22.0 μg/mL).

The reducing SDS-PAGE gels (Figure 5B) showed banding patterns which indicated that the IgG(H)-dsscFv constructs were being expressed correctly with major bands at ~75 kDa and ~25 kDa. Percentage monomer data for wild type and ‘swapped’ IgG(H)-dsscFv proteins is illustrated in the bar graph (Figure 5C) and in Table 4. HER2 IgG displayed a high monomer level (95.6%). In accordance with Fab-dsscFv data, IgG(H)-dsscFv#1 and IgG(H)-dsscFv(FW#2/CDR#1) displayed high monomer levels (92.8% and 94.6%, respectively) whereas IgG(H)-dsscFv#2 displayed a lower monomer level (75.7%). Grafting dsscFv#2 CDRs onto FW#1 resulted in a considerable improvement in monomer level (IgG(H)-dsscFv(FW#1/CDR#2): 96.3% monomer). Thus, it appears that Fv framework ‘swapping’ can be used to enhance the monomer levels of both Fab and IgG-based bispecific antibodies. 

## 4. Discussion

Multimerisation of bispecific antibody formats during cellular protein expression is undesirable and necessitates removal of contaminating multimer. This can result in overall poor yields if the protein is particularly prone to multimerisation. In dsFv/dsscFv-containing bispecifics, the ability of some heavy and light variable domains in separate dsFv/dsscFv monomers to ‘mispair’ during cellular expression may be a reason for <100% monomeric proteins. In isolated scFv, the engineering of a disulphide bond between the vH and vL fixes any multimer formed during cellular expression. Nonetheless, we have previously shown that the disulphide bond is necessary in the final molecule to prevent multimerisation of monomers during and post-purification [2]. The disulphide bond does not on the whole contribute any additional thermal stability nor does it affect the inherent propensity to form monomer/multimer in isolated scFv, at least when placed at the vH44:vL100 position [2]. 

SEC analysis showed that the isolated dsFv and dsscFv in this study were all highly monomeric, although two dsFv appeared to have a low monomer level. In the absence of a linker, it is likely that the lower apparent monomer level is a result of host cell protein co-purification, as opposed to dsFv multimerisation. We show that the monomer level of the isolated dsFv/dsscFv is not predictive of its true multimerisation potential in all protein fusion contexts. There was no correlation between the monomer levels seen in the isolated dsFv with the corresponding Fab-dsFv, and the isolated dsscFv with the corresponding Fab-dsscFv. For example, dsFv#2 and dsFv#3 were ~100% monomeric, but showed vastly different monomer levels when the dsFv was linked to a Fab (Fab-dsFv#3: 83.7% monomer, Fab-dsFv#2: 37.8% monomer). Hence our data suggest that dsFv and dsscFv monomer level screening may be more informative in the context of a relevant scaffold protein. 

We have observed differences in the monomer level between Fab-dsFv and Fab-dsscFv with equivalent sequences in the dsFv/dsscFv position. For sequences that are particularly prone to multimerisation, we have shown that conversion of the dsFv to dsscFv is one potential way to improve the monomer level. In three of the molecules we tested, the monomer level of the Fab-dsFv was high, but was modestly improved by conversion of the dsFv to dsscFv. One of the molecules we tested had a low monomer level as a Fab-dsFv, which was almost doubled upon conversion of the dsFv to dsscFv. 

Differences between Fab-dsFv and Fab-dsscFv monomer levels indicate that linker positioning may be a driving factor for multimerisation. The Fab-dsFv and Fab-dsscFv both contain two linkers. In Fab-dsFv, both variable domains are connected via either a S(G_4_S)_3_ linker or a S(G_4_S/T)_3_ linker to the Fab, whereas in Fab-dsscFv, only one variable domain is connected (via a S(G_4_S)_2_ linker) to the Fab. As both Fv variable domains are coupled to the Fab in Fab-dsFv, longer S(G_4_S)_3_ or S(G_4_S/T)_3_ linkers are necessary in Fab-dsFv to prevent multimerisation induced by linker sterical constraints [4]. In Fab-dsscFv, the dsscFv is coupled to the Fab by only a single linker, therefore there appears to be enough freedom of movement in the dsscFv even with a shorter S(G_4_S)_2_ linker to prevent linker-induced multimerisation. It should be noted that we have previously observed identical monomer levels in Fab-dsscFv regardless of whether the linker between the Fab and dsscFv is 11 amino acids (S(G_4_S)_2_) or 16 amino acids (S(G_4_S)_3_). We have also previously observed identical Fab-dsFv monomer levels, whether the 16 amino acid linker between the Fab and dsFv is (S(G_4_S)_3_) or (S(G_4_S/T)_3_). The Serine to Threonine substitution has been used historically to facilitate cloning of HC/LC double gene vectors, but has no effect on expression or biophysical properties of Fab-dsFv (data not shown). 

Promiscuous v-region pairing appears to be less problematic in Fab-dsscFv, which is likely owing to more efficient intra-molecular dsvH-dsvL pairing as a result of the polypeptide linker connecting the two v-regions. In Fab-dsFv, the dsvH and dsvL are located on separate heavy and light chain polypeptides that must come together within the monomer without the aid of a linker connecting the two domains, whilst evading competition from Fab heavy and light chain interactions or light chain dimer interactions in the ER. 

Conversion of Fab-dsFv to Fab-dsscFv does not always result in efficient monomer formation. Engineering the vH-vL interface in dsscFv so as to alter the specificity, strength or speed of dsvH-dsvL assembly may thus serve to affect correct vH-vL pairing within the dsscFv monomer and thus monomer levels in Fab-dsscFv. Perhaps one way of inadvertently achieving this is through framework ‘swapping’, as we have shown that grafting the dsscFv CDRs from a multimerisation-prone Fab-dsscFv onto a different framework can result in an improved Fab-dsscFv monomer level. Egan et al. [19] have similarly utilised framework swapping to improve scFv monomer level by exchanging the scFv vL framework region IV sequence with that of a corresponding λ-type germline sequence, although the authors utilised non-disulphide stabilised isolated scFv. Framework swapping has also been used to reduce precipitation of an aggregation-prone IgG1 antibody [20]. 

The risk of affinity loss upon conversion of a Fab to scFv might hinder the therapeutic application of scFv in bispecific formats [2]. Formats with appended dsFv are an alternative in situations where conversion to scFv is problematic. The maximal increases in monomer level observed through CDR grafting in this study was 9.3% (Fab-dsFv), 19.2% (Fab-dsscFv) and 19.9% (IgG(H)-dsscFv). It will be interesting to see in future studies whether Fv CDR grafting onto alternative framework sequences can provide even greater increases in monomer level. 

Interestingly, the thermal stability of the Fv domain in Fab-dsFv and Fab-dsscFv formats was largely dependent on the Fv CDRs and largely independent of the Fv framework residues. This is not to say that framework residues do not have any influence over Tm, as previous studies involving CDR grafting or engineering of Fv framework residues have resulted in marked differences in scFv Tm [21,22], although CDRs have also been shown to have strong effects on scFv thermal stability [23,24]. It is not clear from this study what influence, if any, the Fv Tm has on the overall Fab-dsFv/dsscFv monomer level. It is however interesting that in this study, Fab-dsFv/dsscFv with lower Fv Tms (Fv#1 ~ 59 °C; Fv#3 ~ 62 °C) generally displayed higher monomer levels (83.7–91.8%), whilst those with higher Tms (Fv#2 ~ 73 °C; Fv#4 ~ 73 °C) generally displayed lower monomer levels (37.8–79.9%).

In summary, selection of Fv with an inherent propensity to form monomeric hetero-pairs is desirable, and we recommend these are constrained by a disulphide bond for manufacturing preferences [2]. We find that the monomer levels of isolated dsFv/dsscFv cannot be used to predict the monomer levels of formats with appended dsFv/dsscFv. Empirical screening of dsFv or dsscFv in the context of the desired fusion partner is thus advisable for determining the inherent multimerisation propensity of dsFv/dsscFv. We have shown that the monomer level of dsFv-containing bispecific antibody formats can be increased by conversion of the dsFv to dsscFv. For dsscFv sequences that still result in sub-optimal monomer levels, we have shown that grafting the scFv CDRs onto a different framework can further increase the monomer level. We have applied this technique to both Fab- and IgG-appended bispecific antibodies, demonstrating improved monomer levels in both formats. Our framework ‘swapping’ approach can complement existing methods to enhance monomer level, such as CDR mutagenesis, and may be useful where CDR mutagenesis is undesirable in order to maintain precise epitope binding.

In conclusion, we have provided insight into the molecular factors involved in bispecific antibody multimerisation and have demonstrated that there are different driving forces behind dsFv- and dsscFv-mediated multimerisation. These data may be informative to researchers interested in making dsFv/dsscFv-linked Fab or IgG bispecific formats with increased monomer levels.

## Figures and Tables

**Table 1:** Comparison of isolated dsFv and dsscFv. Following the 10-day transient expression in 293F cells, the expression level of dsFv#1–4 and dsscFv#1–4 proteins in the culture supernatant was measured by Octet. Proteins were purified from the supernatant using Ni^2+^-NTA resin, and the purified proteins analysed by S200 SEC and differential scanning fluorimetry.

	dsFv				dsscFv			
	Expression (μg/mL)	μg Final Product/mL Expression Culture	Monomer (%)	Tm (°C)	Expression (μg/mL)	μg Final Product/mL Expression Culture	Monomer (%)	Tm (°C)
#1	<LOD	2.4	44.4 ± 5.8 ^#^	59.5 ± 0.9 *	14.9 ± 1.3	6.4	97.0 ± 3.4	57.8 ± 0.7
#2	29.4 ± 1.3	12.8	100.0 ± 0.0	75.2 ± 0.2	58.3 ± 5.7	27.0	100.0 ± 0.0	76.9 ± 0.4
#3	37.6 ± 0.4	11.7	99.4 ± 1.1	66.8 ± 0.3	47.3 ± 8.7	18.1	99.5 ± 1.0	61.2 ± 0.6
#4	<LOD	1.3	40.3 ± 5.5 ^#^	75.4 ± 0.2 *	15.3 ± 1.4	4.1	93.4 ± 1.2	74.7 ± 0.0

Data shows mean ± SD from three independent transfections. ^#^ Actual monomer level is likely to be ~100%; lower apparent monomer level indicates contamination by host cell proteins. * An additional minor Tm at ~52 °C was observed, which likely represents contaminating host cell proteins. LOD = Limit of detection.

**Table 2:** Comparison of Fab-dsFv vs. Fab-dsscFv formats. Following the 10-day transient expression in 293F cells, the expression level of Fab-dsFv#1–4 and Fab-dsscFv#1–4 proteins in the culture supernatant was measured by a Protein G HPLC assay. Proteins were purified from the supernatant using Protein G HPLC, and the purified proteins analysed by G3000 SEC and differential scanning fluorimetry.

dsFv/dsscFv	Fab-dsFv				Fab-dsscFv			
	Expression (μg/mL)	Monomer (%)	Fab Tm (°C)	dsFv Tm (°C)	Expression (μg/mL)	Monomer (%)	Fab Tm (°C)	dsscFv Tm (°C)
#1	23.4 ± 3.1	87.5 ± 0.5	78.7 ± 0.2	58.4 ± 0.1	20.0 ± 2.9	91.2 ± 0.2	78.8 ± 0.2	59.0 ± 1.2
#2	28.1 ± 2.2	37.8 ± 1.0	78.8 ± 0.0	72.4 ± 0.9	21.7 ± 1.0	70.9 ± 1.6	78.9 ± 0.4	73.1 ± 0.9
#3	24.5.0 ± 2.1	83.7 ± 1.7	78.8 ± 0.2	62.5 ± 0.5	15.8 ± 0.1	91.8 ± 0.5	78.8 ± 0.3	61.4 ± 0.9
#4	15.1 ± 2.6	76.0 ± 0.4	78.8 ± 0.2	73.6 ± 0.3	21.8 ± 3.9	79.9 ± 0.8	79.0 ± 0.4	72.7 ± 0.3

Data shows mean ± SD from three independent transfections.

**Table 3:** Comparison of Fab-dsFv and Fab-dsscFv with wild type and FW/CDR-‘swapped’ Fv. Following the 10-day transient expression in 293F cells, the expression level of wild type and framework ‘swapped’ Fab-dsFv and Fab-dsscFv proteins in the culture supernatant was measured by a Protein G HPLC assay. Proteins were purified from the supernatant using Protein G HPLC, and the purified proteins analysed by G3000 SEC and differential scanning fluorimetry.

dsFv/dsscFv	Fab-dsFv				Fab-dsscFv			
	Expression (μg/mL)	Monomer (%)	Fab Tm (°C)	dsFv Tm (°C)	Expression (μg/m)	Monomer (%)	Fab Tm (°C)	dsscFv Tm (°C)
#1 *	23.4 ± 3.1	87.5 ± 0.5	78.7 ± 0.2	58.4 ± 0.1	20.0 ± 2.9	91.2 ± 0.2	78.8 ± 0.2	59.0 ± 1.2
#2 *	28.1 ± 2.2	37.8 ± 1.0	78.8 ± 0.0	72.4 ± 0.9	21.7 ± 1.0	70.9 ± 1.6	78.9 ± 0.4	73.1 ± 0.9
FW#1/CDR#2	34.0 ± 2.1	47.1 ± 1.3	79.1 ± 0.4	68.4 ± 0.4	28.5 ± 1.4	90.1 ± 0.2	79.1 ± 0.3	72.4 ± 0.2
FW#2/CDR#1	10.6 ± 0.3	91.6 ± 0.4	80.8 ± 0.5	53.7 ± 1.2	6.7 ± 0.6	91.9 ± 0.5	81.5 ± 0.1	53.6 ± 1.4

Data shows mean ± SD from three independent transfections. * Same data as in Table 2, shown here for ease of comparison with FW/CDR ‘swap’ data.

**Table 4:** Comparison of IgG(H)-dsscFv with wild type and FW/CDR-‘swapped’ Fv. Following the 10-day transient expression in 293F cells, the expression level of wild type and framework ‘swapped’ IgG(H)-dsscFv proteins in the culture supernatant was measured by a Protein G HPLC assay. Proteins were purified from the supernatant using Protein A HPLC, and the purified proteins analysed by G3000 SEC.

dsFv/dsscFv	IgG(H)-dsscFv	
	Expression (μg/mL)	Monomer (%)
#1	22.0 ± 2.4	92.8 ± 0.6
#2	24.9 ± 0.6	75.7 ± 2.3
FW#1/CDR#2	39.1 ± 0.6	96.3 ± 1.0
FW#2/CDR#1	3.7 ± 0.4	94.6 ± 0.6

Data shows mean ± SD from three independent transfections.

## Supplementary Materials

The following are available online at http://www.mdpi.com/2073-4468/7/3/35/s1, Figure S1: Gel filtration calibration, Figure S2: Thermograms of purified proteins.
