# Supplementary material for: Relative Contribution of Framework and CDR Regions in Antibody Variable Domains to Multimerisation of Fv- and scFv-Containing Bispecific Antibodies
Source: Antibodies (Basel). 2018 Aug 31;7(3):35. doi: 10.3390/antib7030035 (PMC6640685; doi:10.3390/antib7030035)
Supplement: Supplementary file 1 [file antibodies-07-00035-s001.pdf]

antibodies

MDPI

Supplementary: Relative Contribution of Framework and CDR Regions in Antibody Variable Domains to Multimerisation of Fv- and scFv-Containing Bispecific Antibodies

Pallavi Bhatta * and David P. Humphreys

Protein Sciences Group, UCB Pharma, Slough, Berkshire SL1 3WE, UK; David.Humphreys@ucb.com
* Correspondence: Pallavi.Bhatta@ucb.com

Received: 12 July 2018; Accepted: 30 August 2018; Published: 31 August 2018

Supplementary Data

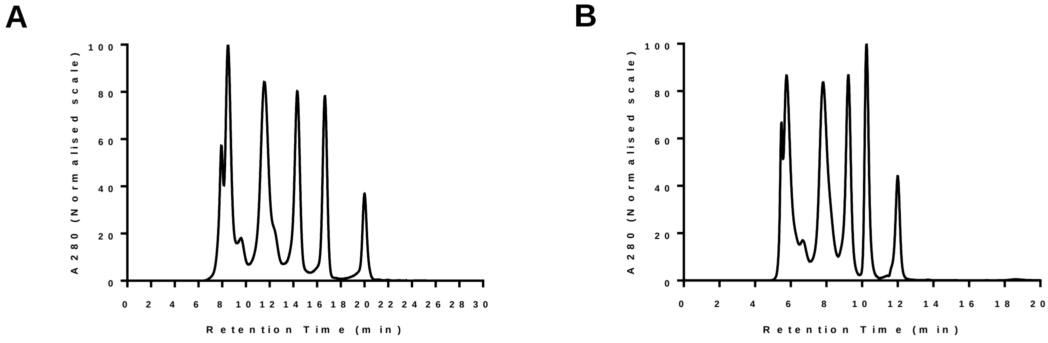

Figure S1. Gel filtration calibration. (**A**) S200 SEC profile. (**B**) G3000 SEC profile. Gel filtration standard (BioRad) was loaded for molecular weight estimation of test samples. The mixture contained bovine thyroglobulin (670,000 Da), bovine γ-globulin (158,000 Da), chicken ovalbumin (44,000 Da), equine myoglobin (17,000 Da) and vitamin B12 (1350 Da).

antibodies

MDPI

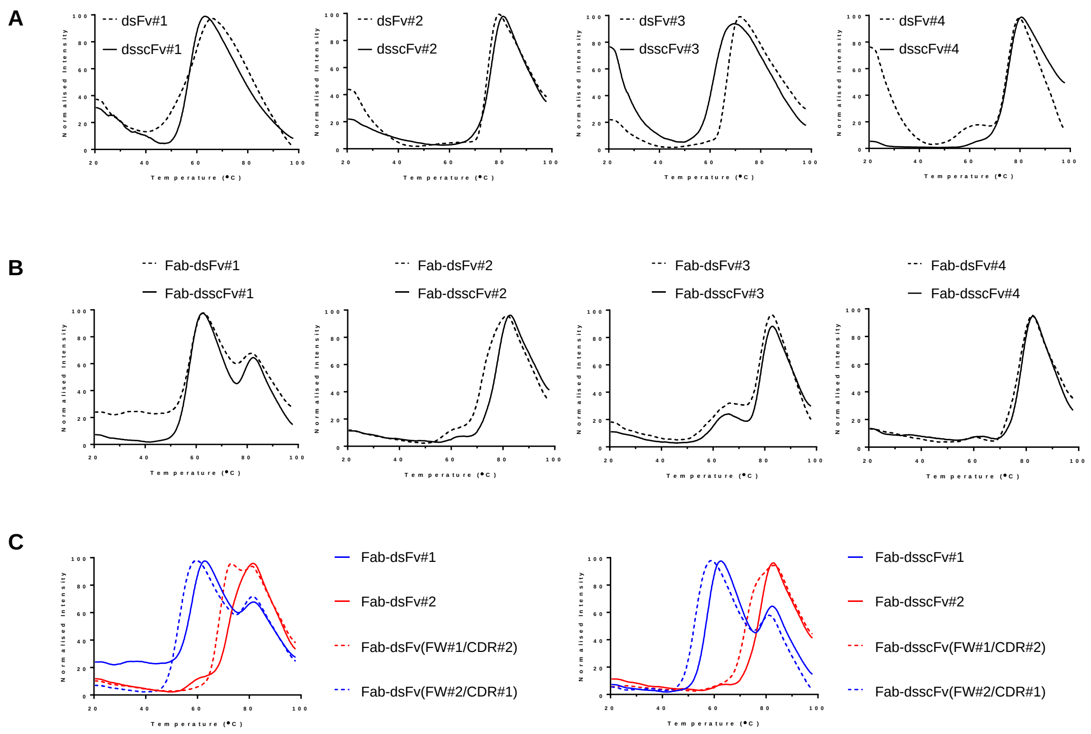

Figure S2. Thermograms of purified proteins. (**A**) dsFv vs dsscFv. (**B**) Fab-dsFv vs Fab-dsscFv. (C) Wild type vs FW/CDR 'swapped' Fab-dsFv and Fab-dsscFv. Fluorescence changes in protein/SYPRO®Orange dye mixtures were analysed on a 7900HT fast real-time PCR System, set at 20 °C to 99 °C with a ramp rate of 1.1 °C/min. The thermograms show unfolding transitions of proteins in PBS pH7.4. The inflection point of the slope(s) was used to generate the T_m.

© 2018 by the authors. Licensee MDPI, Basel, Switzerland. This article is an open access article distributed under the terms and conditions of the Creative Commons Attribution (CC BY) license (http://creativecommons.org/licenses/by/4.0/).
